# The Transplantation of Teeth

**Published:** 1891-09

**Authors:** 


### The Transplantation of Teeth.

The transplantation of teeth is a proceedure, the practical value of which has not as yet been fully established. It is true that a number of cases have been reported in which teeth have been successfully implanted in alveoli from which they have been accidentally removed, or transplanted from one person's jaw to another's, but the permanence of the results thus obtained is still a matter of question. Of course, to secure success from this operation the implanted tooth must contract firm adhesions to the surrounding alveolar structures, and hence it is necessary to investigate closely the processes by which union occurs in these cases.

In a monograph on this subject Dr. Julius Scheff details a large number of experiments made on animals, together with histological examinations, which tend to show that, in the majority of instances the union is periosteal. It may take place by first intention, the alveolar periosteum uniting directly with the cement of the tooth, without absorption of the latter; or the periosteal proliferation may produce a more or less extensive absorption of the cement, which if carried too far may result in extrusion of the tooth. The experiments also demonstrated that the pulp of every implanted tooth becomes necrotic, and this condition may persist, notwithstanding the occurrence of firm periosteal adhesions, or a new tissue may take the place of the necrotic structures. In the dog this new tissue originates in the pulp canals and consists of a delicate vascular connective tissue which differs from the normal pulp by the absence of odontoblasts, or it result from a proliferation of the periosteum into the pulp canals through openings caused by absorption of their walls. If the latter occurs the periosteum may undergo osseous transformation after absorption has ceased.

It would seem from the author's experiments that implanted teeth usually become fixed to the alveolar cavity by firm periosteal adhesions, and that the union is likely to be permanent if the necrotic changes in the pulp have not advanced too far. Of great interest in this connection are the investigations recently made by a Russian physician, Dr. Znameusky, regarding the implantation of artificial teeth both in animals and human beings. He experimented with teeth constructed of porcelain, hard-rubber and metal, at the roots of which he made a large number of fine incisions. The process by which union was brought about consisted in a proliferation of granulation tissue from the alveolar walls into the fine openings at the root of the tooth, thus holding it firmly in position. It is questionable, however, whether artificial teeth can be imbedded with sufficient firmness to withstand, for any length of time, the violence to which they are subjected during mastication.—*International Journal of Surgery.*

